# Review of Tissue-Protective Cytokines

**DOI:** 10.3389/fimmu.2013.00349

**Published:** 2013-12-09

**Authors:** Charles A. Dinarello

**Affiliations:** ^1^Department of Medicine, University of Colorado Denver, Aurora, CO, USA; ^2^Department of Experimental Medicine, Radboud University, Nijmegen, Netherlands

**Keywords:** inflammation, EPO, cytokine biology, erythroid progenitor cells, erythropoietic cytokine, neuroprotective, non-erythropoietic EPO, platelets

Tissue-Protective Cytokines is an up-to date collection of articles edited by Pietro Ghezzi and Anthony Cerami (1). These editors are the leaders in the field, primarily with their pioneering studies on the ability of erythropoietin (EPO) to protect from cell death. The book is unique in many ways. Many chapters (19 Chapters) contain detailed methods and protocols for the experimental models, particularly in the brain and nervous system. This is an added benefit to the book because it helps the reader understand the models being used to evaluate the mechanism as well as the efficacy of tissue-protective cytokines that are being considered for therapies in various diseases. Most of the chapters review published studies or present new data on the properties and mechanism of action of EPO.

As written in the Preface by Cerami, the EPO story starts with well know clinical observation in hemodialysis patients being treated with EPO for low level of hemoglobin. The patients, weak from years of low hemoglobin, feel better much faster and time-wise significantly before there is any increase in red blood cell mass. This suggested that EPO possessed another property. This observation encouraged Cerami, Ghezzi, and their collaborators to look carefully for the effect of EPO in animal models of inflammation. As pointed out in the Preface, Cerami’s studies on the tissue-protective properties of EPO led to the development of novel small molecules that do not stimulate erythropoiesis but rather protect against loss of cell function. The long-term collaboration of Ghezzi and Cerami on the inflammatory properties of tumor necrosis factor (TNFα) allowed for a new collaboration focused on the non-erythrogenic properties of EPO. Ghezzi is a world expert in animal models of inflammation having made many contributions to interleukin-1 (IL-1) and TNF. For example, Ghezzi was the first to report that hypoxia induced IL-1β and TNFα.

In addition to their own chapters, the invited contributions are easy to read and each addresses a different aspect of this growing field of investigation. Driving the field are the clinical trials of EPO in patients with cerebral vascular accidents (stroke) and acute myocardial infarction, in which a benefit was reported. New clinical trials are presently underway. Experimental autoimmune encephalomyelitis (EAE) is the well established model for the human disease Multiple Sclerosis. The therapeutic benefit of EPO in this model is well-described in a chapter by Cerevellini, Ghezzi, and Mengozzi. It seems that this disease as well as ALS are ideally suited for EPO testing.

The chapters provide clear information for both the novice entering the field or the investigator who studies mechanisms of cell death. Needless to write, the book is highly useful to the field of neurodegeneration. The first chapter by Brines and Cerami on the structure-function relationships of the unique, alternate EPO receptor is well written with clear diagrams showing the domains of the heterodimeric EPO receptor that triggers the protective pathway. Other chapters include explanations on EPO and non-EPP tissue targets, the structural basis for other tissue-protective cytokines and the properties of IL-6 as a regenerative cytokine. The chapter on IL-6 is highly relevant as this cytokine, often regarded as a pro-inflammatory cytokine, is often a protective cytokine. Galun and Stefan-Rose-John present their studies on Hyper IL-6 which delivers a potent signal for liver regeneration. What remains unclear is why IL-6 blockade with anti-IL-6 receptor is effective in treating rheumatoid arthritis. If IL-6 signaling induces regeneration, the treatment of rheumatoid arthritis patients with anti-IL-6 R should reduce joint integrity. One is left with the conundrum that the efficacy of anti-IL-6 R in rheumatoid arthritis is due to a reduction in B-cell function more than any anti-inflammatory property.

There are several chapters that discuss how EPO protects against brain and spinal cord injury. Related to the neuroprotective properties of EPO, there is an interesting chapter on rodent behavior as related to depression. This area of investigation, that is, cytokines that affect depression, is not restricted to rodent experiments. Indeed, Kevin Tracey has been a leader in the area of brain cytokines. In fact, for many years we know that patients with rheumatoid arthritis receiving an intravenous infusion of anti-TNFα antibodies have a near immediate improvement in sense of well-being which is unrelated to any effect of TNFα blockade peripherally, and there are reports that anti-TNFα is an anti-depressant. Since EPO crosses the blood brain barrier, one could easily conclude from reading these chapters that EPO could be used as an anti-depressant. Indeed, small molecule EPO mimetic analogs, such as ARA 290, which is active orally, and that triggers the tissue-protective, anti-inflammatory effects of EPO, could be used to treat depression.

Related to this field is a chapter on the methods for studying pharmacologic treatment of post-traumatic neuropathic pain. Also related to this area is the observation that EPO can be used to reduce the peripheral neuropathy that develops in rats with type 1 diabetes.

In addition to the intellectual excellence of this book, many chapters contain detail explanations with diagrams in the various models, particular in studies on nerves and the brain.

No study on the anti-inflammatory properties of a tissue-protective cytokines such as EPO would be complete without a study using the model of acute myocardial infarction in mice. The chapter by Talan and Latini is excellent. This chapter contains a detailed review of EPO’s ability to reduce infarct size in animal models of total left anterior coronary artery occlusion as well as models of ischemic-re-perfusion. There is also a list of clinical trials with EPO administered at various time points following acute myocardial infarction. Most studies on the list result in a non-significant effect on the primary end-point with one exception. An Italian study in patients treated with EPO within 6 h of the infarction showed a statistically significant reduction in the infarct size as determined by level of enzymes and also a statistically significant increase in left ventricular ejection fraction at 6 months following an initial dose of EPO at soon after the infarct and a second dose at 24 and 48 h. This trial, like the stroke trials are essentially why the field will grow and mature.

This book, the first of its kind, will serve a vital function for those entering the field.
